# Predictors of Non-Obstructive Coronary Artery Disease in Patients Undergoing Elective Coronary Angiography

**DOI:** 10.5334/gh.1204

**Published:** 2023-05-11

**Authors:** Ghazal Peerwani, Saba Aijaz, Sana Sheikh, Salim S. Virani, Asad Pathan

**Affiliations:** 1 Department of Clinical Research Cardiology, Tabba Heart Institute, Karachi, Pakistan; 2 Department of Clinical Cardiology, Tabba Heart Institute, Karachi, Pakistan; 3 The Aga Khan University, Karachi, Pakistan, Texas Heart Institute, Houston, TX, USA

**Keywords:** Non-obstructive coronary artery disease, coronary angiogram, non-invasive testing, Pakistan, Low-middle-income-country

## Abstract

**Background:**

Appropriate patient selection for coronary angiography (CAG) is essential to minimize the unnecessary risk of morbidities and exposure to radiation and iodinated contrast. This becomes even more relevant in low-to-middle-income settings where most health expenditures are out-of-pocket due to lack of medical insurance. We determined predictors of non-obstructive coronaries (NOC) in patients undergoing elective CAG.

**Methods:**

CathPCI Registry®, single-center data was extracted for 25,472 patients who had CAG over an eight year period. After excluding patients for compelling conditions or known CAD, 2,984 (11.7%) patients were included in this study. Non-Obstructive Coronaries was defined as <50% left main coronary artery and major epicardial vessel stenosis. Multiple Cox proportional algorithm was employed to report prevalence ratios (PR) of predictors of NOC along with 95% confidence interval.

**Results:**

Mean age of patients was 57.9 ± 9.7 years, 23.5% were women. Preprocedural non-invasive testing (NIT) was performed in 46% of the patients; of which 95.5% reported to be positive but only 67.3% were stratified as high risk. Of 2,984 patients undergoing elective CAG, 711 (24%) had NOC. Predictors of NOC included younger age <50 years (PR: 1.3, CI: 1.0–1.5), Women (1.8, 1.5–2.1), low (1.9, 1.5–2.5) and intermediate risk stratification (1.3, 1.0–1.6) on Modified Framingham Risk Score and inappropriate (2.7, 1.6–4.3) and uncertain (1.3, 1.1–1.6) classification of CAG on Appropriate Use Criteria. Patients with heart failure as an indication of CAG (1.7, 1.4–2.0) and No NIT or positive low risk NIT (1.8, 1.5–2.2) were more likely to have NOC.

**Conclusion:**

Approximately one out of four patients undergoing elective CAG had NOC. Yield of diagnostic catheterization can be improved by adjudicating NIT especially in younger patients, women, patients with heart failure as an indication of CAG, patients classified as inappropriate on Appropriate Use Criteria and patients categorized as low or intermediate risk on MFRS.

## Introduction

Coronary angiography (CAG) is a key diagnostic test to identify patients with obstructive coronary artery disease (CAD) [1] and decide which patients will benefit from coronary revascularization. However, CAG is an invasive test and is associated with a significant cost to the individual patient due to the associated procedural risk [2] as well as to the healthcare system. Appropriate patient selection is crucial to limit the number of patients with no significant coronary obstruction [3]. The rates of normal or non-obstructive coronaries (NOC) have been suggested as a performance measure in the cardiac catheterization laboratory [4]. Utilization of pre-test probability, Modified Framingham Risk score (MFRS), Appropriate Use Criteria (AUC) for diagnostic catheterization, and non-invasive testing (NIT), can be some of the ways to achieve optimal patient selection for invasive CAG [5,6,7].

Although definitions vary, angiographically normal or NOC finding has been reported in 21% to 58.4% of patients undergoing CAG for the diagnosis of CAD in a non-emergency setting [6,7,8,9,10]. Predictors associated with NOC in prior studies included women, age, absence of insulin-dependent diabetes, absence of dyslipidemia, atypical ischemic symptoms or asymptomatic presentation, absence of peripheral vascular disease, and negative or positive low-risk NIT results and low and intermediate MFRS [7,8]. Unfortunately, very limited data is available from low to middle-income country (LMIC) settings on the frequency or predictors of NOC on CAG.

In Western world data, the number of NOC findings has raised concerns. The implication of this becomes even more relevant in a LMICsetup with an enormous aggregate global burden of CAD and a self-pay environment due to lack of medical insurance or national health care systems. Due to cost constraints, NIT may be under-utilized by the physicians, and additionally, the AUC for CAG, at best, remains unused in these setups. Moreover, unnecessary risk of morbidities and exposure to radiations and contrasts might also be avoided if predictors of NOC are identified and considered during patient selection. The rationale for this study is to explore patterns and predict factors associated with NOC in resource-limited settings in order to develop an evidence base to aid clinicians in deciding when to employ CAG. Thus this study determines the frequency and predictors of NOC in patients undergoing elective CAG.

## Methods

A cross-sectional study design based on a retrospective record review was employed to determine the frequency and predictors of NOC in patients who underwent elective CAG in a tertiary care setup of Tabba Heart Institute (THI), Karachi, Pakistan, during the span of June 2012 to July 2020. CathPCI Registry^®^ data associated with National Cardiovascular Data Registry (NCDR)^®^ was extracted for 24,472 patients. We excluded patients with compelling indications for CAG, including acute coronary syndrome (ACS), cardiogenic shock, pulmonary edema, known CAD (prior coronary revascularization or myocardial infarction), and prerequisite elective CAG for valve replacement surgery.

Patients’ baseline demographic data, such as age and gender, cardiovascular risk factors history, clinical presentation, medications history, other relevant cardiac test findings, and angiographic findings, were extracted from the electronic medical records. Patients presenting symptoms were categorized as: typical, angina without change in frequency or pattern for the past six weeks and is controlled by rest or oral or transcutaneous nitroglycerine; atypical/unlikely ischemia, pain or discomfort in chest, neck, or arms, not necessarily or exertion and not consistent with the pain of myocardial ischemia, likely due to non-cardiac conditions; and asymptomatic, no angina symptoms [11].

Non-invasive testing included exercise electrocardiogram stress test (ETT), stress echocardiogram (SE), myocardial perfusion imaging (MPI), and Coronary CT angiogram (CCTA). Non-invasive testing results were taken as positive, negative, or indeterminate. In addition, risk stratification of positive NIT into high, intermediate, and low risk was done. In patients with a positive stress test, a Duke Treadmill score of ≥5 was taken as low risk, a score between –10 and +4 as intermediate risk, and a score ≤–11 as high risk. In patients who underwent SE, normal stress echocardiographic wall motion or no change of limiting wall motion abnormalities was taken as low risk, peak segmental wall motion index (peak WMSI) of 1.1 to <1.7 as intermediate, and peak 1.7 or more as high risk. In patients who underwent MPI, summed stress score (SSS) of 4 to 7 was taken as low risk, SSS of 8 to 12 as intermediate risk, and SSS of 13 or higher as high risk.

MFRS was computed using parameters of age, sex, smoking status, dyslipidemia, hypertension, and diabetes. As the data was unavailable for parameters, like total cholesterol and high density lipoprotein, the score was imputed as 2 points if the patient had dyslipidemia or was taking statins. Likewise, for unavailability of parameters like systolic blood pressure and the number of medications for hypertension, the score was imputed as 2 points for a man with a history of hypertension or was taking anti-hypertensives and 3 points for a woman with a history of hypertension or was taking anti-hypertensives. The computed score was categorized as low risk (<10%), intermediate-risk (10–19%), and high risk (>20%) [12,13].

Appropriate use criteria for diagnostic catheterization was calculated based on the severity of angina, use of anti-ischemic therapy, and NIT findings. Based on the above parameters, each cardiac catheterization procedure was assigned an appropriateness score from 1 to 9. A score of 7 to 9 denoted the patient as an appropriate indication for CAG, a score of 4 to 6 denoted uncertain indication, whereas a score of 1 to 3 denoted inappropriate indication [14].

Non-obstructive coronaries was defined as <50% in the left main (LM) coronary artery and major epicardial vessels [15,16]. Stenosis of ≥50% was termed as moderately or severely obstructive coronaries (MoSOC). The degree of stenosis on angiographic findings was documented as per the interpretation by the performing cardiologist. Less than 50% stenosis is not recorded based on the most recent data collection form for the NCDR CathPCI Registry, so we cannot separate normal from mildly obstructive coronaries.

Mean and standard deviations were reported for quantitative variables and frequency and percentages for categorical variables as a part of descriptive analysis. In addition, the frequency and percentages of NOC were reported. Baseline and procedural characteristics were compared between NOC and MoSOC. The cox-proportional algorithm was employed for univariate and multivariable modeling. Adjusted prevalence ratios (PR) (significance level <0.05) of predictors of NOC, along with 95% confidence intervals (CI), were reported. All statistical analyses were performed using STATA version 16.

Approval was taken from THI Institutionalized Review Board (IRB) (THI/IRB/SQ/11-12-2020/082). THI IRB granted a waiver of informed consent and exemption for this study.

## Results

**Figure 1:**
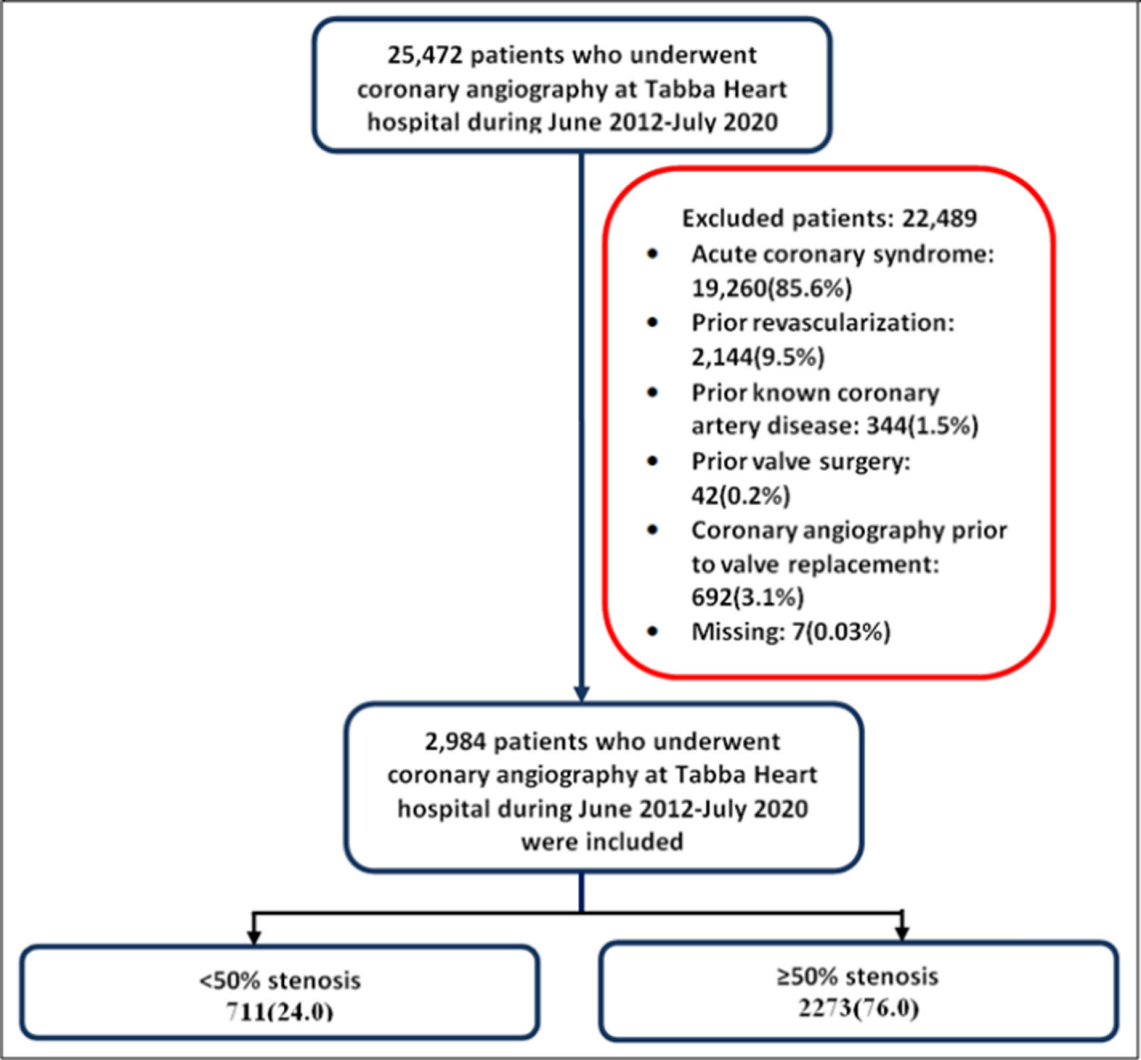
Study flow diagram.

Out of 25,472 patients who underwent CAG at the THI cath lab over eight years, 2,984 (11.7 %) were analyzed in our study after applying the exclusion criteria (Figure 1).

### Coronary angiography findings

Overall of 2,984 patients undergoing elective CAG, 24% (n = 711) had NOC. Out of patients with MoSOC ≥ 50% stenosis) (n = 2273, 76%), 31.6% had a single vessel, 28.8% had two vessels coronary artery disease (2 VCAD) and 39.6% had three vessels coronary artery disease (3 VCAD). Significant LM disease was present in 7% of patients.

### Baseline characteristics as per coronary angiography findings

In the NOC group, patients were more likely to be younger <50 years (28.4% vs. 16.1%) (55.2 ± 10.2 vs. 58.8 ± 9.4 years) compared to the MoSOC group. Also, more women were likely to have NOC (39.9% vs. 18.3%). Diabetes was less common in patients with NOC (42.3% vs. 55.8%) compared to MoSOC patients. A lower proportion of patients underwent CAG due to positive stress tests (25.8% vs. 53.3%) and the presence of chest pain (without positive NIT) (23.4% vs. 26.8%) in the NOC group compared to the No MoSOC group (Supplemental table 1). Also, patients with NOC were more likely to present with atypical/unlikely angina (20.4% vs. 7.4%) or were asymptomatic at presentation (38.7% vs. 21.9%). More patients were categorized as low (48.4% vs. 28.6%) or intermediate risk (36.3% vs. 44.4%) on MFRS in the NOC group compared to the no MoSOC group (Table 1).

**Table 1:** Characteristics of patients as per coronary angiography findings.


CHARACTERISTICS	TOTAL n = 2984 n(%)	NON-OBSTRUCTIVE CORONARIES n = 711(24.0)	MODERATELY OR SEVERELY OBSTRUCTIVE CORONARIES n = 2273(76.0)	p-VALUE

Age*	57.9 ± 9.7	55.2 ± 10.2	58.8 ± 9.4	<0.001

**Age <50**	567(19)	202(28.4)	365(16.1)	<0.001

**Women**	701(23.5)	284(39.9)	417(18.3)	<0.001

BMI*~	27.8 ± 4.9	28.5 ± 6.0	27.6 ± 4.5	NS^+^

**Prior heart failure**	115(3.9)	41(5.8)	74(3.3)	0.01

**Dyslipidemia**	1052(35.3)	203(28.6)	849(37.4)	<0.001

**Hypertension**	2054(68.8)	467(65.7)	1587(69.8)	NS

**Family history of premature CAD**	501(16.8)	108(15.2)	393(17.3)	NS

**Diabetes mellitus**	1569(52.6)	300(42.2)	1269(55.8)	<0.001

**Smoker**	433(14.5)	77(10.8)	356(15.7)	0.003

**Indication**				

Positive stress test	1396(46.8)	184(25.8)	1212(53.3)	<0.001

Heart failure evaluation	697(23.4)	303(42.6)	394(17.3)	

Angina	774(25.9)	167(23.4)	607(26.8)	

Other	117(3.9)	57(8.2)	60(2.6)	

**Symptoms**				

Typical angina	1898(63.5)	291(40.9)	1,607(70.7)	<0.001

Atypical angina/unlikely angina	313(10.5)	145(20.4)	168(7.4)	

Asymptomatic	773(25.9)	275(38.7)	498(21.9)	

**Modified Framingham risk score**				

Low	950(31.8)	344(48.4)	606(26.6)	<0.001

Intermediate	1,268(42.5)	258(36.3)	1,010(44.4)	

High	766(25.7)	109(15.3)	657(29.0)	

**Operators as per annual volume**				

>400	1397(46.8)	278(39.1)	1,119(49.2)	0.001

100–400	1,170(39.2)	313(44.0)	857(37.7)	

<100	417(14.0)	120(16.9)	297(13.1)	


* Mean ± standard deviation. ~ BMI is weight in kg divided by the square of height in meters Chi-sq test used to determine p-values. + Independent sample T-test used to determine the p-value CAD: Coronary artery disease, NS: Nonsignficant.

### Non-invasive stress testing

**Figure 2:**
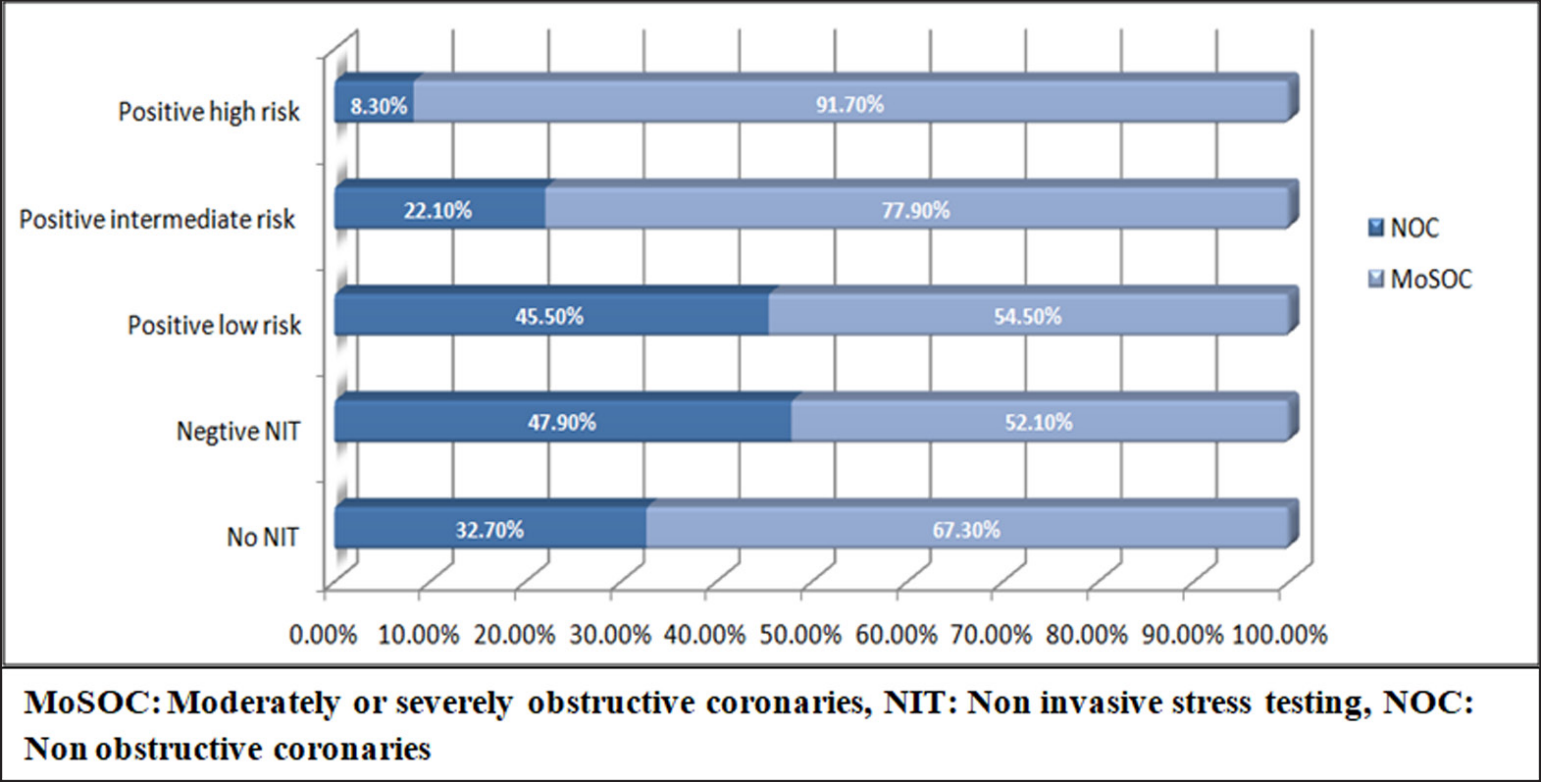
CAG findings as per NIT results.

NIT was performed in 49% of the patients prior to CAG. Of patients who didn’t undergo any NIT, 32.7% had NOC. Abnormal or positive NIT result was reported in 95.5% of patients who underwent NIT. The majority of patients with positive NIT results had MoSOC (86.8%). Most patients with positive high-risk NIT findings had MoSOC (91.7%) (Figure 2, Table 2).

**Table 2:** Findings of Non-invasive testing as per coronary angiography findings (n = 1462).


	N(%)	NON-OBSTRUCTIVE CORONARIES n = 711(24.0)	MODERATELY OR SEVERELY OBSTRUCTIVE CORONARIES n = 2273(76.0)	p-VALUE

**No NIT**	1522(51)	497(32.7)	1025(67.3)	<0.001

**NIT**	1462(49)	214(14.6)	1248(85.4)	

**Type of NIT**				

**ETT**	452(30.9)	68(15)	384(85)	

Low risk	14(3.1)	7(50)	7(50)	

Intermediate	164(36.3)	32(19.5)	132(80.5)	

High risk	265(58.6)	34(12.8)	231(87.2)	

**SE**	175(11.9)	46(26.3)	129(73.7)	

Low risk	2(1.1)	1(50)	1(50)	<0.001

Intermediate	50(28.6)	25(50)	25(50)	

High risk	113(64.6)	16(14.2)	97(85.8)	

**SPECT-MPI**	796(54.5)	94(11.8)	702(88.2)	

Low risk	17(2.1)	8(47.1)	9(52.9)	

Intermediate	189(23.7)	36(19.1)	153(80.9)	

High risk	540(67.8)	28(5.2)	512(94.8)	

**CCTA**	39(2.7)	6(15.4)	33(84.6)	

**Results of NIT**				

Positive	1396(95.5)	184(13.2)	1212(86.8)	<0.001

Negative	48(3.3)	23(47.9)	25(52.1)	

Indeterminate	18(1.2)	7(38.9)	11(61.1)	

**Risk stratification of NIT**				

Low	33(2.4)	15(45.5)	18(54.5)	<0.001

Intermediate	403(29.5)	89(22.1)	314(77.9)	

High	918(67.3)	76(8.3)	842(91.7)	

Unavailable	10(0.7)	2(20)	8(80)	


* The percentages in columns of non-obstructive coronaries and moderately or severely obstructive coronaries are row percentages to provide a % of moderately or severely and non-obstructive coronaries as per non-invasive test finding. CCTA: Cardiac computed tomography angiography, ETT: Standard exercise test, NIT: non-invasive testing, SE: Stress echocardiogram, SPECT-MPI: Single photon emission computed tomography-Myocardial perfusion imaging.

### Appropriate use criteria

**Figure 3:**
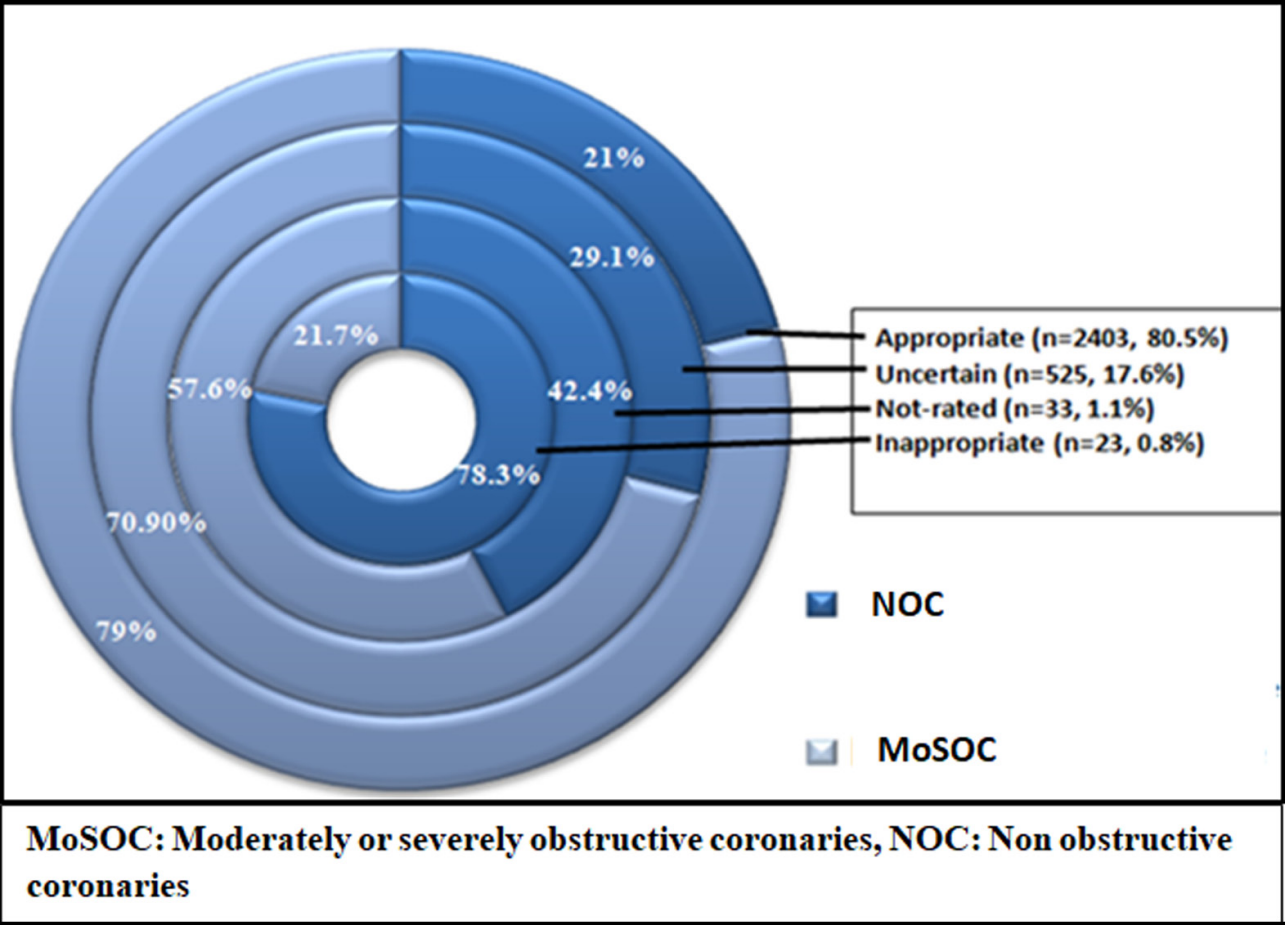
Bull’s eye diagram depicting CAG as per AUC stratification.

In almost 80.5% (n = 2403) of the patients, CAG was classified as appropriate, followed by 17.6% (n = 525) of patients in whom CAG was categorized as uncertain. Patients classified as appropriate on AUC were less likely to have NOC (21% vs. 79%) (Figure 3).

### Predictors of non-obstructive coronary artery disease

The multivariable Cox proportional algorithm results indicated younger age <50, women, heart failure as an indication of CAG, inappropriate stratification on AUC, low and intermediate MFRS, and no NIT or positive low-risk NIT as significant predictors of NOC (Table 3)

**Table 3:** Predictors of non-obstructive coronaries in patients undergoing elective coronary angiography.


CHARACTERISTICS	CRUDE PREVALENCE RATIOS (95% CI)	ADJUSTED PREVALENCE RATIOS (95% CI)	p-VALUE

**Age < 50**	1.7(1.4–2)	1.3(1.0–1.5)	0.03

**Women**	2.1(1.9–2.5)	1.8(1.5–2.1)	<0.001

**Modified Framingham risk score**			

Low	2.5(2.1–3.1)	1.9(1.5–2.5)	0.02

Intermediate	1.4(1.1–1.7)	1.3(1.0–1.6)	

**Appropriate use criteria**			

Inappropriate	3.6(2.2–5.7)	2.7(1.6–4.3)	0.02

Uncertain	1.3(1.1–1.6)	1.3(1.1–1.6)	

Not rated	1.9(1.1–3.3)	1.4(0.8–2.5)	

**No NIT testing or positive low-risk NIT findings**	2.6(2.2–3.1)	1.8(1.5–2.2)	<0.001

**Indication for CAG**			

Heart failure/LV dysfunction	2.4(2.1–2.8)	1.7(1.4–2.0)	<0.001


* Variables including prior heart failure, diabetes, dyslipidemia, smoking history, CAD presentation, and operator as per annual volume were not found significant in the multivariable model. ** Older age ≥ 50, men, high MFRS, appropriate AUC, positive intermediate/high-risk NIT, and indications other than heart failure/LV dysfunction were taken as reference. CAG: coronary angiography, LV dysfunction: left ventricular dysfunction, NIT: non-invasive testing.

### Temporal trends from 2012–2020

**Figure 4:**
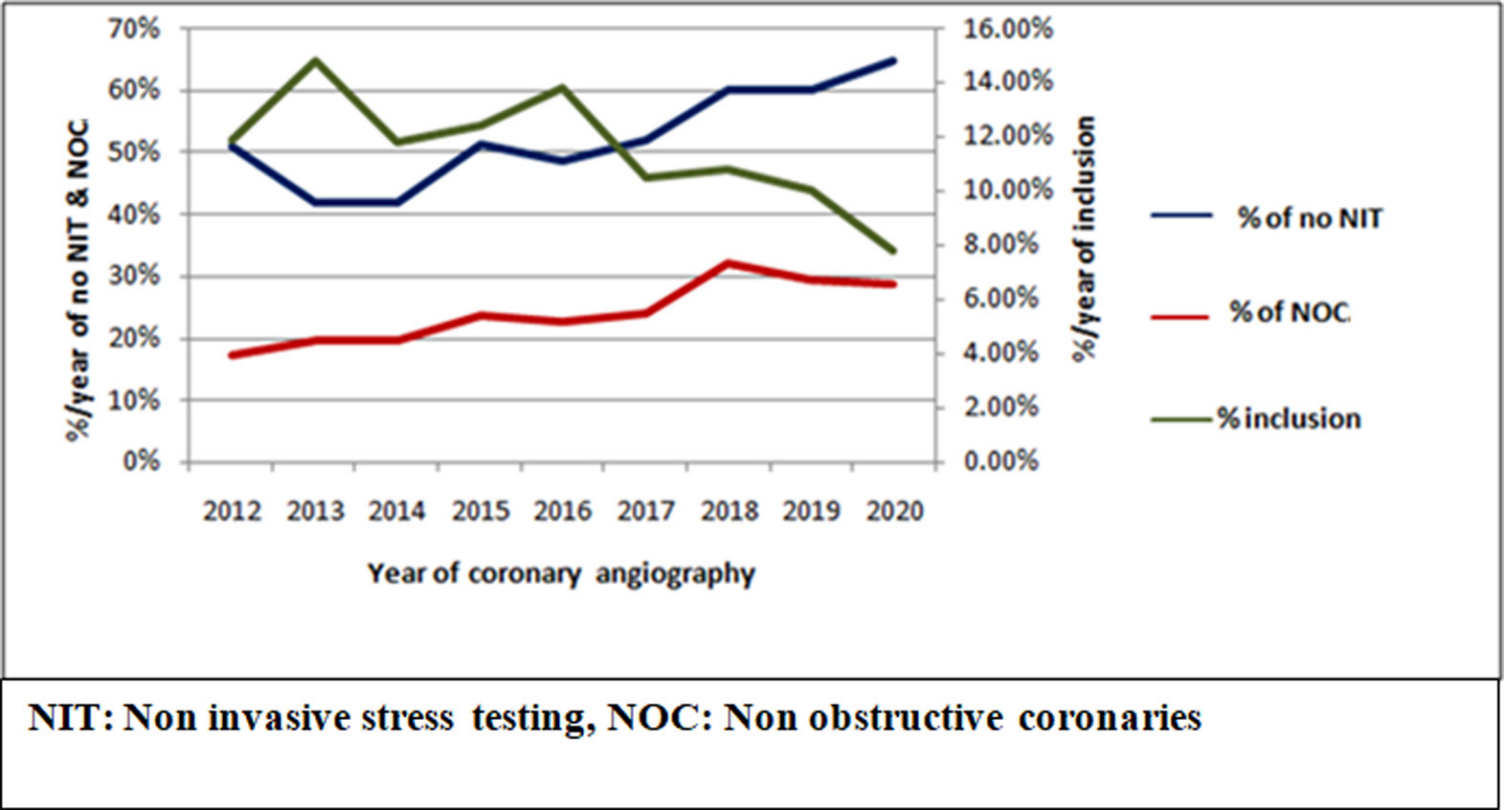
Temporal trends of inclusion of elective coronary angiography patients, NOC and NIT during 2012–2020.

**Figure 5:**
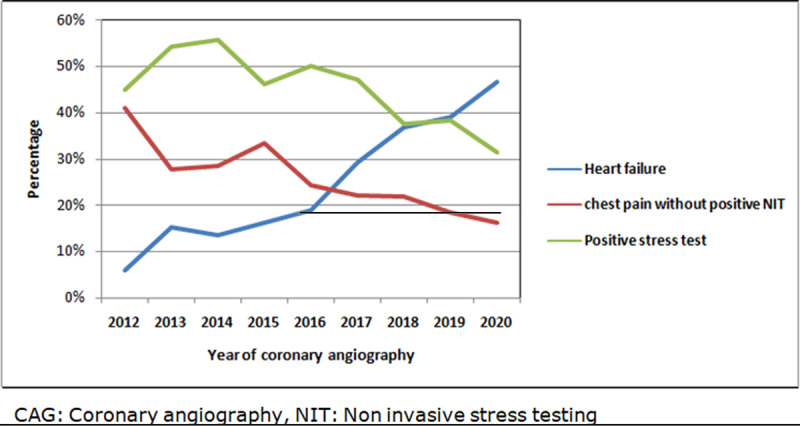
Temporal trends for indications of elective CAG during 2012–2020.

Figure 4 depicts a significantly decreasing trend in the inclusion of elective CAG patients from 2012–2020. Simultaneously, there was a significantly increasing trend of NOC and decreasing trend of perfoming NIT over an eight year period. Figure 5 depicts the trends of indications of CAG during eight years. There was an increasing trend of heart failure as an indication of elective CAG whereas there was a significantly decreasing trend of positive stress test and chest pain without positive stress test as indications of elective CAG during 2012–2020.

## Discussion

In this single-center registry from an LMIC spanning an eight year period, among the entire group of patients undergoing CAG, in a large majority (>75%) of patients, the procedure was performed for urgent or emergent indications. Elective CAG in patients without known CAD comprised only 11.7% of the overall diagnostic CAG volumes; of these 2,984 patients, 24% had NOC. Significant predictors of NOC were younger age, female, heart failure indication for the procedure, low and intermediate-risk MFRS, no NIT or positive low-risk NIT findings, and being categorized as inappropriate on AUC for CAG.

Coronary angiography is an essential diagnostic test, the role of which in patients with suspected stable ischemic heart disease is to define the presence and pattern of obstructive CAD and help select patients who may benefit from coronary revascularization in addition to background optimal medical therapy. In our study, the proportion of patients without known CAD who underwent non-emergency CAG is much smaller than in other reports. Our study had an 11.7% inclusion rate compared to approximately 21% in the NCDR registry and data from Ontario and VA-CART, with an inclusion rate of 31% and 25%, respectively [6,8,9]. Additionally, in our study, the vast majority (>75%) underwent CAG for the indication of the ACS. Known CAD (10%) and other indications comprised a very small component. This pattern is very different from what has been reported from North America. In the NCDR report, 26% had ACS as the underlying diagnosis, and 41% had known CAD [8]. Similarly, the VA-CART and Ontario Cardiac Registry had 13–22% of patients with ACS and 33–47% with prior cardiac history [6,9]. Pakistan is a developing country with limited healthcare resources. This influences health-seeking behaviors and service utilization of the Pakistani population, which varies vastly from developed countries. Health behaviors stem from socio-economic determinants, cultural and political context, and health literacy levels [17,18]. Similar to most LMIC, in our population, seeking professional help for health conditions is usually delayed until there is urgency or symptoms present themselves. We also found a decreasing trend over the study duration in the percentage of elective CAG patients included. We don’t have a good explanation for this, except perhaps there has been greater emphasis on the invasive treatment of ACS patients over the last decade. Due to single center data, patients referred to other regional emerging centers with angiography labs may have increased leading to lower referral to our center.

We found obstructive CAD (>70% stenosis) in over 70% of patients without known coronary disease undergoing elective CAG. The frequency of obstructive CAD has ranged from 30% to 52% in the different North American registries, including the overall 38% from the large NCDR data [7]. Similarly, NOC has been much higher, ranging between 39.2% to 58.4 [7,8]. This suggests a higher threshold for CAG in our practice setting despite the fact that there is a fee-for-service model at our institution, which may incentivize doing more procedures. We found a high yield for critical disease represented by significant (LM) disease in 7%, 39.6% with 3 VCAD, and 43.4% LM or 3VCAD, a finding that is three to five fold higher than the 7 to 13% LM or 3 VCAD reported from New York state or Ontario registries [19]. This makes it less likely that a restricted approach to CAG is associated with the under-detection of patients with the critical disease. However, conclusively establishing this would require national mortality registries and individual level outcome data that are missing in LMICs such as Pakistan.

We also found an overall increase in the frequency of NOC findings over the study duration which was accompanied by more patients undergoing CAG with indication of heart failure due to left ventricular systolic dysfunction. This subset makes up nearly half of all the patients enrolled. This finding further endorses one of our previously published data on patients that, with unexplained left ventricular systolic dysfunction as the indication for CAG, 58.8% had any MoSOC, very similar to the 56.5% in the current larger data set [20]. Although this is an appropriate indication for CAG according to the ACC/AHA AUC for CAG [14], other testing modalities such as coronary artery calcium score alone or in combination with CCTA can be considered. Even in these patients, the power of zero calcium score is still valid with an associated <1% likelihood of obstructive CAD [21], requiring no further coronary evaluation. Among patients with any coronary calcium, a CCTA can be considered if they have a low-moderate coronary calcium burden and high likelihood of good quality CT angiogram. In the remaining patients, directly proceeding with an invasive CAG will be appropriate. Such strategy will increase the yield of invasive CAG and, at the same time, be associated with greater patient safety [22].

Non-invasive testing was performed in 49% of our study patients with a decreasing trend over the study duration, comparatively lower than NIT performed in the North American registries (59%–84%) [6,7,8,9]. This can largely be explained by the greater inclusion of patients for heart failure with reduced ejection fraction evaluation, most of whom did not have a stress test prior to CAG. However, risk stratification and referral for CAG in our study are markedly different from the NCDR results [8], although there is a lack of data on patients who underwent NIT but were not referred for CAG, and assumptions can only be made based on the patient group who underwent CAG. In our study, 2/3 of the NIT had high-risk positive results and 2.4% had low risk positive stress test compared to 5.3% and 31.9% respectively in the NCDR report [8]. We found that the yield of positive stress tests with subsequent detection of obstructive CAD is remarkably different from North American real-world results with 13.2% having non-obstructive coronaries vs 53.4% in the NCDR report and 8.3% vs 32.5% among patients with high-risk stress test findings; SPECT imaging performing better than exercise ECG and stress echo modalities [8]. The poor yield and the failure to achieve the gatekeeper role by NIT in the real-world US clinical practice has brought into question the patient selection for stress testing, the quality of the tests being done as well the actual performance of these tests [23]. A need for better stress tests has been brought up by experts with the current tests felt to have only marginal utility. However, our results are contrary to this with the stress testing modalities retaining their risk stratification role and 92.6% having MoSOC after an intermediate or high-risk positive NIT. Difference in patient selection for NIT with less use in low risk and asymptomatic individuals, different quality control measures, less ambiguous interpretation of borderline results or artifacts in SPECT imaging, more restrictive referral for CAG after abnormal results may be some of the reasons. Clearly, the decision-making process for CAG in our study setting is very different from the reports from US and Canada [8,9]. What should be an acceptable number of the NOCAD in elective CAG is unclear. Even in ACS or STEMI patients up to 10% can have NOC [24]. Whether our results of NOC are too low and we are causing harm by missing patients who are appropriately referred for this test, remains unestablished. Here in, use of coronary calcium scoring and CCT may potentially be very helpful in risk stratifying such patients. Among patients with borderline abnormal NIT, a calcium score of zero may more likely suggest the absence of obstructive CAD [25]. Likewise, in patients with a low-intermediate positive NIT and low-moderate coronary calcium burden, the use of CCTA can be an effective non-invasive risk stratification tool and function well as a gatekeeper [26,27].

Our study findings indicated that women, younger age, absence of symptoms, or atypical angina were predictors of NOC. Moreover, low and intermediate risk on MFRS and inappropriate for CAG on AUC were also factors associated with NOC. Additionally, NOC was also associated with positive low-risk NIT findings or no NIT. These predictors are consistent with the published literature [7,8,9]. Although the inappropriate rating on AUC is a predictor of NOC, the overall clinical utility of AUC in identifying obstructive CAD is of very limited clinical value as less than 2% of study subjects were in inappropriate or not rated categories.

Overall, as compared to US results, our study patients are younger, less likely to be a woman, smoker, having dyslipidemia, obesity, or family history of premature CAD, and twice likely to have diabetes. These findings are very consistent with prior published reports where patients of South Asian origin tend to get the coronary disease at a younger age with two-fold higher diabetes prevalence and are less likely to have other traditional risk factors for CAD [28,29]. Current risk calculators tend to underestimate risk in South Asians even though they tend to have a higher coronary calcium and atherosclerosis disease burden on CCTA. There are biological and non-biological pathways that contribute to the enhanced risk of coronary atherosclerosis in South Asians. Factors such as insulin resistance, metabolic syndrome, type 2 diabetes, obesity, dyslipidemia including elevated lipoprotein (a), hypertension, chronic kidney disease, enhanced inflammation and thrombosis, enhanced genetic susceptibility are compounded by diet, physical inactivity, tobacco products, and social, psychological, and environmental factors [28]. Future studies are required to understand genetic and pharmacogenetic differences in South Asians and to allow precision medicine efforts.

To the best of our knowledge, this is the first study reporting frequency and predictors of NOC in a LMIC setting. The sample is also one of the study’s strengths as it represents the population reporting to tertiary care hospitals for elective CAG.

Our study has limitations. First, it is a retrospective single-center study, where interpretation of NIT is done by a cardiologist certified in nuclear cardiology or stress echocardiography with an active peer-review process. Second, analysis of CAG findings is limited to subjective individual physician interpretation. Moreover, due to our database format, percentage of stenosis is reported only for those patients with more than 50% stenosis, hence there is an inability to differentiate between near normal and non-obstructive findings. Third, MFRS might have underestimated the risk of CAD in our study because we imputed values for blood pressure and lipid levels. This was evident from our results as MFRS classified 25.7% of the study population as high risk whereas the 76% had CAD, out of which 28.8% and 39.6% had 2 VCAD and 3 VCAD. It is one of the limitations of our study that the data is just from a single center, therefore the geographical variations of Pakistani population couldn’t be assessed. Lastly, NIT risk stratification couldn’t be assessed for half of the patients included in this study, and we only have the outcome of patients who underwent CAG after abnormal stress test. There is also a lack of data on patients who did not undergo CAG after normal or abnormal NIT results.

## Conclusion

In our current clinical practice, one in four patients undergoing elective CAG had NOCAD. The number of NOC have increased over an eight year period. Despite the guideline, only 49% of the patients underwent NIT. Preprocedural NIT performed well in risk stratification with a very high yield of obstructive CAD. Yield of CAG can be vastly improved by adjudicating alternate modalities like NIT especially in younger patients, women, patients with heart failure as an indication of CAG, and patients classified as low or intermediate risk on MFRS. Additionally, utility of coronary calcium score and CCTA can also be explored to improve patient selection and preprocedure assessment of disease likelihood.
